# Using joint models to study the association between CD4 count and the risk of death in TB/HIV data

**DOI:** 10.1186/s12874-022-01775-7

**Published:** 2022-11-18

**Authors:** Nobuhle N. Mchunu, Henry G. Mwambi, Dimitris Rizopoulos, Tarylee Reddy, Nonhlanhla Yende-Zuma

**Affiliations:** 1 Biostatistics Unit, South African Medical Research Council (SAMRC), SAMRC Building, 491 Peter Mokaba Ridge Road, Durban, 4041 South Africa; 2 University of KwaZulu-Natal, School of Mathematics, Statistics and Computer Science, King Edward Avenue, Pietermaritzburg, 3209 South Africa; 3 Centre for the AIDS Programme of Research in South Africa (CAPRISA), University of KwaZulu-Natal, 719 Umbilo Road, Durban, 4041 South Africa; 4 Department of Biostatistics, Erasmus University Medical Center, Rotterdam, CE 3000 The Netherlands; 5 Department of Epidemiology, Erasmus University Medical Center, Rotterdam, CE 3000 The Netherlands; 6 MRC-CAPRISA HIV-TB Pathogenesis and Treatment Research Unit, Doris Duke Medical Research Institute, University of KwaZulu-Natal, Durban, South Africa

**Keywords:** Time-to-event data, Longitudinal data, Joint models, CD4 count, Mortality, Association structures

## Abstract

**Background:**

The association structure linking the longitudinal and survival sub-models is of fundamental importance in the joint modeling framework and the choice of this structure should be made based on the clinical background of the study. However, this information may not always be accessible and rationale for selecting this association structure has received relatively little attention in the literature. To this end, we aim to explore four alternative functional forms of the association structure between the CD4 count and the risk of death and provide rationale for selecting the optimal association structure for our data. We also aim to compare the results obtained from the joint model to those obtained from the time-varying Cox model.

**Methods:**

We used data from the Centre for the AIDS Programme of Research in South Africa (CAPRISA) AIDS Treatment programme, the Starting Antiretroviral Therapy at Three Points in Tuberculosis (SAPiT) study, an open-label, three armed randomised, controlled trial between June 2005 and July 2010 (N=642). In our analysis, we combined the early and late integrated arms and compared results to the sequential arm. We utilized the Deviance Information Criterion (DIC) to select the final model with the best structure, with smaller values indicating better model adjustments to the data.

**Results:**

Patient characteristics were similar across the study arms. Combined integrated therapy arms had a reduction of 55% in mortality (HR:0.45, 95% CI:0.28-0.72) compared to the sequential therapy arm. The joint model with a cumulative effects functional form was chosen as the best association structure. In particular, our joint model found that the area under the longitudinal profile of CD4 count was strongly associated with a 21% reduction in mortality (HR:0.79, 95% CI:0.72-0.86). Where as results from the time-varying Cox model showed a 19% reduction in mortality (HR:0.81, 95% CI:0.77-0.84).

**Conclusions:**

In this paper we have shown that the “current value” association structure is not always the best structure that expresses the correct relationship between the outcomes in all settings, which is why it is crucial to explore alternative clinically meaningful association structures that links the longitudinal and survival processes.

## Background

In HIV studies, researchers are mostly interested in the association between CD4 counts (or viral loads) and time to AIDS or death where both the association structure of repeated biomarkers and primary survival endpoints are studied [1, 2]. Classical models such as time-varying Cox models [3] as well as separate analysis comprising of linear mixed effects (LME) models for longitudinal data [4], and Cox proportional hazard (PH) models [5] have been traditionally used to study the association between time-dependent covariates and the hazard of an event [5–8]. However, separate analysis may not be appropriate for such data because they fail to account for the dependency and the association between the longitudinal and survival processes, resulting in biased estimates [2]. An alternative would be to use the time-varying Cox model [3], where the longitudinal measurements are directly incorporated into the Cox model. However, this method uses the last-observation-carried-forward (LOCF) approach which is not a realistic approach for clinical biomarkers such as CD4 counts since they evolve dynamically with time and are of endogenous nature [9]. In the literature, it has been shown that using the time-varying Cox model for endogeneous covariates by treating them as exogeneous covariates in the model produces spurious results [9, 10].

A powerful method that takes into account the dependency and association between longitudinal and time-to-event outcomes is joint models of longitudinal and time-to-event data [9, 11]. Formally, a joint model consists of two linked sub-models, where a linear mixed effect model is commonly used for the longitudinal sub-model, and the Cox proportional hazards model is often used for the survival sub-model. The association structure linking the two sub-models is of fundamental importance in the joint modelling framework and the choice of this structure should be made based on the clinical background of the study. However, this information may not always be accessible. Rationale for selecting this association structure has been proposed in the literature.

Within the standard joint model formulation, the “current value” association structure is widely used because it is simple and has a clear interpretation of the relationship between the longitudinal and survival processes. However, it may not be realistic to expect that it will always be the most appropriate functional form in expressing the correct association between the outcomes in all settings. This is because, in general, there could be other characteristics of the subjects’ longitudinal profiles that are more strongly predictive for the risk of an event and the consideration of competing association structures to describe the link between the two processes is very important.

In light of this, our main aim is to explore alternative functional forms of the association structure that links the two processes that have been proposed in the literature [12, 13] and we mainly focus on the four most frequently used specifications of the assocation structure in the joint modeling literature [14–16] that allow the rate of increase/decrease of the longitudinal outcome or a suitable summary of the whole longitudinal trajectory to determine the risk for an event. We discuss their formulations and how to select the best structure in the subsequent sections. To illustrate the virtues of joint modeling, we also aim to compare the results obtained from the joint model to those obtained from the time-varying Cox model.

## Methods

### Source of data and description

We used data from the Centre for the AIDS Programme of Research in South Africa (CAPRISA) AIDS Treatment programme, the Starting Antiretroviral Therapy at Three Points in Tuberculosis (SAPiT) study, an open-label, three arm randomized, controlled trial between 28 June 2005 and 04 July 2010. The trial was designed to determine the optimal time to initiate ART in patients with HIV and TB co-infection who were receiving TB therapy [17, 18]. More details about the study and the results for primary and secondary outcomes have been published in detail elsewhere [17–20].

### Statistical analysis

Descriptive data, was presented as means with standard deviations (SD) or medians with interquartile range (IQR) and percentages. We fitted ten different mixed effects models for the longitudinal sub-model and the Bayesian Information Criterion (BIC), was used to select the best model, where smaller values are preferable. To extend the standard joint model, we considered four functional forms of the association structure and used the Deviance Information Criterion (DIC) to select the final joint model, with smaller values indicating better model adjustments to the data. All multivariable models were adjusted for the study arm (combined early and late integrated therapy arms versus sequential therapy arm as in the primary paper [17]), gender and age. A square root transformation was used to normalize the CD4 count. Analyses were conducted using SAS, version 9.4 (SAS Institute INC., Cary) and R version 4.1.2. The most recent JMbayes package [21] was used to fit the joint models and assess different association structures. We used a Bayesian estimation procedure and a Markov chain Monte Carlo (MCMC) algorithm to fit the joint models because of its flexibility in dealing with complex models.

### The joint model formulation

To formulate a standard joint modelling framework, we follow the typical setup where a mixed-effects model is used for the longitudinal data and a Cox proportional hazards (PH) model is used for the time-to-event data, with the two models sharing some random effects. This is the so called shared parameter model approach [9, 22–24].

#### The longitudinal sub-model

**Fig. 1:**
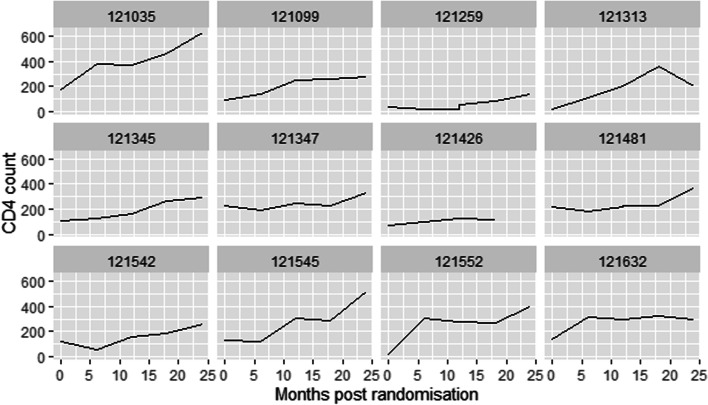
Individual longitudinal profiles of CD4 count over time

**Fig. 2:**
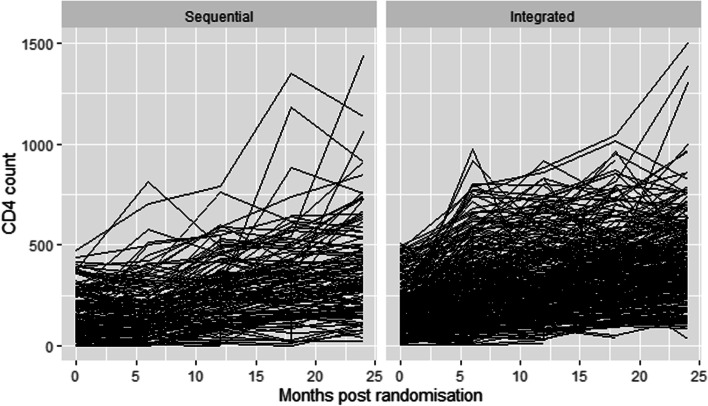
Spaghetti plots by study arm

To measure the effect of the longitudinal covariate to the risk for an event, $m_{i} (t)$ needs to be estimated and successfully reconstruct the complete longitudinal history for each subject. In order for this to work a suitable mixed-effects model is postulated to describe the subject-specific time evolutions. Results in Fig. 1 shows an apparent non-linearity of the subject-specific square root CD4 count profiles for 12 randomly selected individuals and is consistent with the spaghetti plots (Fig. 2). This suggests that a non linear mixed-effects model could be a plausible starting point [25]. Thus, we fitted ten different mixed effects models and used the BIC to select the best model for our data and the results reveal that the inclusion of only the natural cubic splines of time in both the fixed and random effects parts of the longitudinal sub-model for CD4 count is preferred. Therefore, the longitudinal sub-model for the $i^{th}$ subject, $i = 1,\cdots , n$ is defined as:

1 $$\begin{aligned} y_{i}(t)= & {} (\beta _{0}+b_{i0})+(\beta _{1}+b_{i1})B_{n}(t,\lambda _{1})\nonumber \\&+(\beta _{2}+b_{i2})B_{n}(t,\lambda _{2})+\epsilon _{i}(t) \end{aligned}$$

where $\left\{ B_{n}(t,\lambda _{k}) : k = 1, 2\right\}$ denotes the B-spline basis matrix for a natural cubic spline of time with one internal knot placed at the 50th percentile for the follow-up times, $\epsilon _{i}(t) \sim N(0, \mathbf {R}_{i})$ and $\mathbf {b}_{i}\sim N(\mathbf {0},\mathbf {D})$, with $\mathbf {R}_{i}=\sigma ^{2}_{\epsilon }\mathbf {I}_{ni}$ and $\mathbf {D}$ an unstructured variance-covariance matrix.

#### The survival sub-model

The main effects of age, gender and study arm are included in the survival sub-model. More specifically, the survival sub-model for the $i^{th}$ individual, $i = 1,\cdots ,n$ using the current value specification is given by:

2 $$\begin{aligned} h_{i}(t)= & {} h_{0}(t)\exp \left\{ \gamma _{1}Arm_{i}+\gamma _{2}Age_{i}+\gamma _{3}Gender_{i}\right. \nonumber \\&\left. +\alpha m_{i}(t)\right\} , \qquad t>0 \end{aligned}$$

where $\gamma ^{T}=\left\{ \gamma _{1},\gamma _{2},\gamma _{3}\right\}$ denote the regression coefficients and $\alpha$ quantifies the strength of the association between the two processes. The baseline hazard function is specified using B-splines, where the logarithm of the baseline hazard is formulated as:

3 $$\begin{aligned} \log (h_{0}(t))=k_{0}+\sum \limits _{q=1}^{Q}k_{q}B_{q}(t,g) \end{aligned}$$

where $k^{T} = (k_{0},\cdots ,k_{Q})$ are the spline coefficients, *g* denotes the degree of the B-splines basis matrix for natural cubic spline of time such that $B_{q}(\cdot ): q=1, \cdots ,Q$, and $Q=\ddot{Q}$ is the number of interior knots [26].

### Alternative association structures

The current value association structure, described in model  2, assumes that for individual *i*, the true value of the longitudinal marker $m_{i}(t)$ at time *t*, is predictive of the risk of experiencing the event at that particular time point *t*. Although this is a simple and a very appealing parameterization providing a clear interpretation for $\alpha$, it assumes that the strength of association between the longitudinal value and the event risk is the same across all individuals, however the hazard may vary across individuals as a function of the other subject-specific covariates, thus this may not be the most optimal association structure to link the two processes. There exist various and more complex parameterizations of the association structure which extend the current value association structure for the survival sub-model proposed in the literature [9, 11, 16, 24, 27], and they can be seen as special cases of the following general formulation of the relative risk model:

4 $$\begin{aligned} h_{i}(t)=h_{0}(t)\exp \left[ \gamma ^{T}w_{i1}+f\left\{ m_{i}(t-c),b_{i},w_{i2};\alpha \right\} \right] , \end{aligned}$$

where $f(\cdot )$ denotes the function of the true level of the marker $m_{i}(\cdot )$, of the random effects $b_{i}$ and extra covariates $w_{i2}$ and describes different functional forms of association between longitudinal and the time to event outcomes. In this paper we discuss four most frequently used association structures in the joint modeling framework presented below;

#### Time-dependent slopes

The current value association structure can be extended by incorporating the rate of change of the true longitudinal trajectory, especially when the direction and strength of trend of a biomarker are as important as its level at a particular time point *t*, the survival sub-model then becomes,

5 $$\begin{aligned} h_{i}(t)=h_{0}(t)\exp \left\{ \gamma ^{T}w_{i}+ \alpha _{1} m_{i}(t)+\alpha _{2} m^{'}_{i}(t)\right\} , \end{aligned}$$

where $m^{'}_{i}(t)=\frac{d}{dt}m_{i}(t)=\frac{d}{dt}\left\{ x^{T}_{i}(t)\beta +z^{T}_{i}(t)b_{i}\right\}$. This parameterization was proposed by [9, 13, 28] and has some beneficial features in the HIV context [29]. The interpretation of parameter $\alpha _{1}$ remains the same as in the standard parameterization (model  2). The parameter $\alpha _{2}$ measures the association between the velocity of the true longitudinal trajectory at time *t* and the risk for an event at the same time point, provided that $m_{i}(t)$ remains constant.

#### Cumulative effects (area)

This structure allows the whole trajectory of the longitudinal marker to be associated with the hazard for an event by including in the linear predictor of the survival sub-model the integral of the longitudinal trajectory, representing the cumulative effect of the longitudinal outcome up to time point *t* [13], this is represented by

6 $$\begin{aligned} h_{i}(t)=h_{0}(t)\exp \left\{ \gamma ^{T}w_{i}+ \alpha \int _{0}^{t} m_{i}(s)ds\right\} , \end{aligned}$$

where for any particular time point *t*, $\alpha$ measures the strength of the association between the risk for an event at time point *t* and the area under the longitudinal trajectory up to the same time *t*, with the area under the longitudinal trajectory regarded as a suitable summary of the whole trajectory [9]. This parametarization have been shown to increase the statistical power of the analyses [30].

#### Weighted cumulative effects (weighted area)

The cumulative effect defined in model  6 assumes that all measurements for a variable from the beginning of the study until time *t* are of equal importance. This may be an unreasonable assumption for studies with longer follow-up periods [31]. Recent values of a measurement closer to the event may be expected to have a higher relevance to the hazard, especially in the case of internal variables in humans [32]. To remedy this a weight function is used in the integral that is a decreasing function of time, such that as time increases, measurements are given less importance. The normal density function is an appropriate choice for assigning weight to values at time $s,\forall s \le t$ [31, 32]. For differential weights in the cumulative effect formulation we get:

7 $$\begin{aligned} h_{i}(t)=h_{0}(t)\exp \left\{ \gamma ^{T}w_{i}(t)+ \alpha \int _{0}^{t} \varpi (t-s)_{+}m_{i}(s)ds\right\} , \end{aligned}$$

where $\varpi (\cdot )$ is an appropriately chosen weight function that places different weights at different time points, with $(t-s)_{+}=t-s$ when $t > s$ and zero otherwise, and $t-s$ denotes time elapsed since exposure, *s* denotes a time prior to or equal to *t*. The standardized normal weight function considered by [31] is given by

8 $$\begin{aligned} \varpi (t-s)_{+}=\frac{\frac{1}{\sigma \sqrt{2\pi }}\exp -\left\{ (t-s)_{+}\right\} ^{2}/2\sigma ^{2}}{\int _{0}^{t.max}\frac{1}{\sigma \sqrt{2\pi }}\exp -\left\{ x\right\} ^{2}/2\sigma ^{2}dx} \end{aligned}$$

The parameter $\sigma$ controls the rate of change in the weights over time. In the papers by [30, 32] the values of parameters were chosen a priori for specific, clinically relevant values of $t-s$. Operating under a Bayesian framework, [31] estimate the value of the scale parameter ($\sigma$) directly from the data, by doing so, this could shed light on the important question of how much of the biomarker’s history do we really need to predict future events, we also take the same approach in this paper. For full details on the formulation and estimation of the weighted cumulative effect parameterization under a Bayesian paradigm, refer to [31].

Several additional association structures that were not explored in this paper have been proposed in the literature [12–16]. These include among others, the “lagged effects”, the “interaction effects” and the “random effects”. The lagged effects parametrization postulates that, the hazard of experiencing the event at time *t* is associated with the level of the longitudinal measure at a previous time point $t-c$, hence with a $c-lag$. Unlike the current value parametarization, the interaction effects association structure allow for different values of association for different patient subgroups and this can be achieved by including interactions between the baseline covariates and the true unobserved longitudinal trajectory function, as a linear predictor in the relative risk model. The random effects parametarization which postulates that patients who have a lower/higher level for the longitudinal outcome at baseline (i.e., intercept) or who show a steeper increase/ decrease in their longitudinal trajectories (i.e., slope) are more likely to experience the event, for more details refer to [33].

### Selecting the best association structure

To select the best functional form for the association structure for our data we make use of the deviance information criterion (DIC), which is a standard approach for model comparison within the Bayesian framework, and it is a very popular model selection criterion when assessing models with posterior distributions obtained through Markov Chain Monte Carlo (MCMC) [34]. Moreover, the DIC is more appropriate for assessing model fit among a set of non-nested candidate models similar in concept to the Akaike information criterion (AIC) and the Bayesian information criterion (BIC) [34]. Suppose that the observed data *y* with unknown parameters $\theta$ has a density $p(y|\theta )$ and deviance $D(\theta )=-2\log \left\{ p(y|\theta )\right\} +C$, where *C* is a constant. A measure of the effective number of parameters as defined by [34] is given by

9 $$\begin{aligned} p_{D}=E_{\theta |y}\left[ -2\log \left\{ p(y|\theta )\right\} +2\log \left\{ p(y|\hat{\theta }(y))\right\} \right] , \end{aligned}$$

where $\hat{\theta }=E[y|\theta ]=\bar{\theta }$, the DIC is then defined as

10 $$\begin{aligned} DIC=D(\bar{\theta })+2p_{D} \end{aligned}$$

and is a combination of the deviance (*D*) and the complexity $(p_{D})$ of the model. Similarly to the AIC and BIC, smaller DIC values mean a better model fit.

## Results

### Exploratory data analyis

**Fig. 3:**
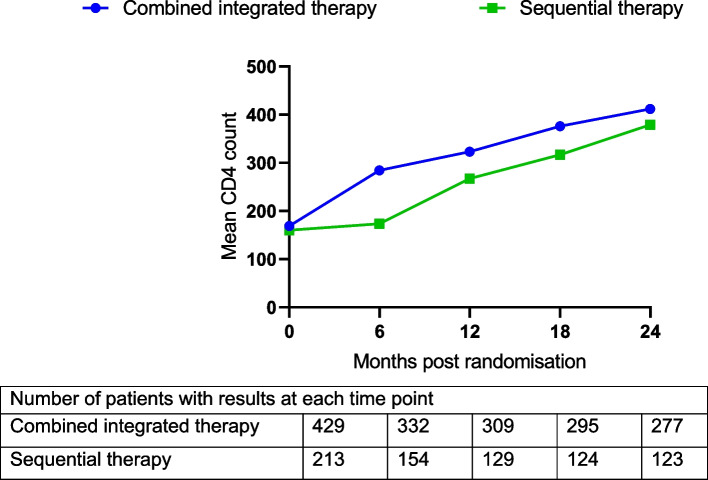
Mean CD4$+$ count (cells/mm$^{3}$) over time

Among the 642 patients enrolled in the SAPiT trial, 429 (66.8%) were in the combined integrated therapy arms and 213 (33.2%) in the sequential therapy arm. Out of the 642, only 501 (78.0%) patients were initiated on ART. The mean age across arms was 34.2 years, and 49.7% of the participants were males. The median CD4 count was 150 and 140 in the combined integrated therapy arms and sequential therapy arm, respectively (Table 1). Patient characteristics were similar across the study arms (Table 1). Figure 3 shows a constantly increasing trend in CD4 count in the combined integrated arms months post randomisation. Contrary to the sequential arm where ART was initiated 6 months post randomisation.

**Table 1:** Baseline characteristics of the study population, stratified by arm

Variable	Combined integrated therapy arms N=429	Sequential therapy arm N=213
Mean age (SD), years	34.4 (8.4)	33.9 (8.2)
Median CD4+ count (IQR), cells/mm$^{3}$	150 (77-254)	140.0 (69-247)
Number of males, n (%)	209 (48.7)	110 (51.6)

### Survival analysis

**Fig. 4:**
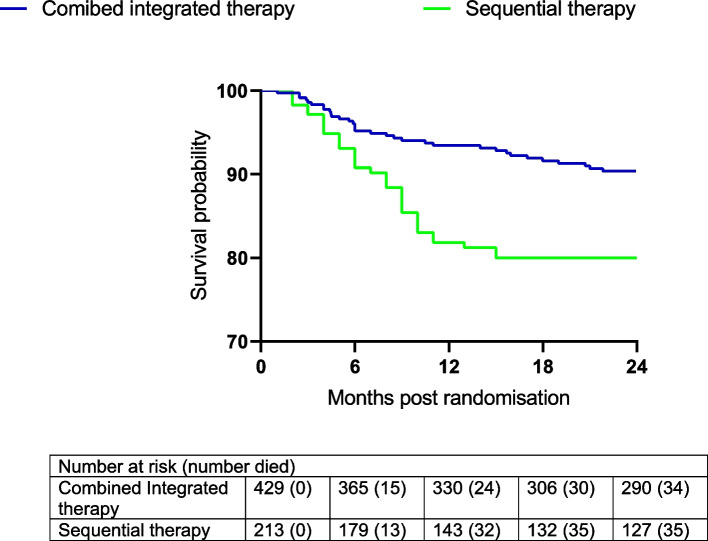
Kaplan-Meier curve for survival

During follow-up, a total of 69 (10.7%) patients died (combined integrated therapy arms (*n*=34) and sequential therapy arm (*n*=35)) and most of these deaths occurred in the first 12 months after randomisation across all arms (Fig. 4). In addition, over 984.79 person-years of follow-up, the mortality rates were 10.1 per 100 person-years (py) (95% confidence interval (CI): 5.9-16.1) in the combined integrated therapy arms and 11.3 per 100 py (95% CI: 7.9-15.8) in the sequential arm. Notably, the mortality rate in the sequential therapy arm was more than double that of the combined integrated therapy arms (Hazard Ratio (HR): 0.45, 95% CI: 0.28-0.72, log rank *p*-value = 0.006, Fig. 4).

### Time-varying Cox model analysis

The results from the time-varying Cox model shows a strong association between the longitudinal CD4 count and the risk of death. In particular a unit increase in the square root CD4 count corresponds to a 19% reduction in the risk of death (HR: 0.81, 95% CI: 0.77-0.84, standard error (SE): 0.023, *p*-value $<0.001$).

### Joint modeling analysis

Based on the DIC values presented in Table 2, there is strong evidence to favour the joint model with cumulative effects parameterization (DIC=21727.38). The results from Table 3 suggests that a higher CD4 count is strongly associated with a significant reduction in the hazard of death. In particular, the joint model finds a significantly strong association between the area under the longitudinal profile of CD4 count and the risk for death, with a unit increase in square root CD4 count corresponding to a 21% reduction in mortality (HR:0.79, 95% CI:0.72-0.86, SE: 0.005).

**Table 2:** Measures of fit for the four different association structures

Association type	df	LPML	DIC	pD
Current value	1961	-11412.23	21742.74	1458.60
Cumulative effects (Area)	1961	-11443.00	21727.38	1426.77
Weighted cumulative effects (Weighted area)	1962	-11474.02	22007.05	1600.78
Time-dependent slopes	1962	-11726.33	22235.78	1457.14

**Table 3:** Hazard ratios and 95% credibility intervals (CI) for the parameters of the survival sub-models from four different joint models

Variable	Current value HR (95% CI)	Area HR (95% CI)	Weighted area HR (95% CI)	Time-dependent slopes HR (95% CI)
Age	0.99 (0.96-1.02)	0.98 (0.96-1.01)	0.99 (0.96-1.02)	0.99 (0.96-1.02)
Integrated therapy	0.68 (0.41-1.12)	0.60 (0.37-0.98)	0.67 (0.40-1.17)	0.63 (0.39-1.01)
Women	0.59 (0.36-0.99)	0.55 (0.33-0.94)	0.57 (0.33-0.97)	0.58 (0.34-0.94)
$\alpha _{1}$	0.82 (0.77-0.88)	0.79 (0.72-0.86)	0.82 (0.77-0.87)	0.82 (0.76-0.87)
$\alpha _{2}$				1.05 (0.74-1.56)

## Discussion

Joint modeling of longitudinal and time to event data is a useful and efficient approach for evaluating associations between the longitudinal CD4 count and the risk of death. In this paper, we sought to explore and discuss alternative functional forms of the association structure between the CD4 count and the risk of death and ultimately select the best form for our data. We found the cumulative effects parameterization to be the best specification for the association structure for our data. The results from this association structure suggested that the area under the longitudinal profile of CD4 count is strongly associated with a significant reduction in the hazard of death. These results are clinically advantageous, as they allow for the calculation of hazard ratios between patients by utilizing their whole longitudinal profile rather than only using their “current value”. In addition, our results gave ample insights into the underlying nature of associations between CD4 count and the risk of death and confirms results from previous studies [35–38] and are consistent with previous results from the same study [39, 40]. Of note, the cumulative effects parameterization have been shown to increase the statistical power of the analyses [30].

Our second aim was to illustrate the virtues of joint modeling by comparing our results to those obtained from the time-varying Cox model. Our joint model found that higher square root CD4 count over time was associated with a 21% reduction in the risk of death, whereas the time varying Cox model found that a higher square root CD4 count over time was associated with a 19% reduction in the risk of death. In addition, compared to the time-varying Cox model, our joint model produced smaller standard errors, indicating an increased efficiency in the joint model [41]. These results are consistent with previous research [42, 43].

According to [44], the Cox model tends to underestimate the true association size of markers due to measurement error. Moreover, joint models are more robust when assessing the association between longitudinal endogenous covariates with time-to-event outcomes [45, 46] and it has been shown elsewhere through various simulation studies in the literature [2, 9, 28, 46–48] that joint models produce unbiased and more efficient estimates of the treatment effects on the time to event and the longitudinal marker, and reduce bias in the estimates of the overall treatment effect [2, 49]. This is particularly important when designing clinical trials, where greater efficiency implies higher power and smaller sample sizes.

## Conclusion

The current value association structure is not always the most optimal form to link the longitudinal and survival processes, in this paper we showed the importance of exploring alternative functional forms of the association structure in order to choose the most optimal association structure to better assess the relationship between the longitudinal and survival process. We hope this information will provide public health researchers with an understanding of what this powerful methodology can offer in substantive terms and how to apply it to their data and also enable them to make better informed decision when choosing the best association structure to link the longitudinal and survival processes. We also hope that this paper will encourage adoption of the joint longitudinal-time-to-event modeling framework in research domains where it is currently underutilized and that should benefit from it.

## Data Availability

The data that support the findings of this study are available from CAPRISA but restrictions apply to the availability of these data, which were used under license for the current study, and so are not publicly available. Data are however available from Professor Kogieleum Naidoo who is a principal investigator of the SAPIT study (Kogie.naidoo@caprisa.org).
